# Affective Changes During Cognitive Behavioural Therapy–As Measured by PANAS

**DOI:** 10.2174/1745017901713010115

**Published:** 2017-08-25

**Authors:** Lars Saxon, Sophie Henriksson, Adam Kvarnström, Arto J. Hiltunen

**Affiliations:** Department of Social and Psychological Studies, Section of Psychology, Karlstad University, Karlstad, Sweden

**Keywords:** Affect, Affective personality, Cognitive behavioural therapy, PANAS, Psychopathology, Hypotheses

## Abstract

**Background:**

Previous researches have indicated that self-reported positive affect and negative affect is changing in a healthy direction during Cognitive Behavioural Therapy (CBT).

**Objective:**

The aim of the present study was to examine how affective personality is related to psychopathology before and after CBT.

**Method:**

A group of clients (n = 73) was measured before and after CBT, differentiated by their problem areas at pre-therapy (i.e., depressive, anxious and mixed).

**Results:**

After therapy, clients experienced higher positive affect (p < .02, d=0.66), lower negative affect (p < .001, d=0.98) and there was a significant change in the distribution of affective personality regardless of problem area, χ^2^ = 8.41, df = 3, two-tailed p = .04, 99% CI [0.03, 0.04]. The change in the distribution was largest for the two most relevant personality types, self-actualization and self-destructive affective personality.

**Conclusion:**

Results indicate that CBT can achieve changes in affect and affective personality.

## 1 INTRODUCTION

In a previous exploratory study, self-reported Positive Affect (PA) and Negative Affect (NA) were studied in clients recruited before, during or after (i.e., from three different phases of) Cognitive Behavioural Therapy (CBT), using a cross-sectional design [1]. The results showed that subjects reported different levels of PA and NA depending on phase of therapy. To further investigate these effects, the present study examined a group of clients before and after CBT (i.e. using a within-subject design), differentiated by their problem areas at pre-therapy.

## 1.1 Affect

In the communities of psychology and psychiatry there has, in the recent years, been an increase in the interest in affects (emotional responses) [2]. This interest, both in research and clinically, has focused on two major directions that both find support depending on what circumstances they are studied in [2]. One direction is biologically grounded with a focus on the study and description of separate affects [3]. The second direction has a broader interest describing affects in terms of dimensions [4, 5]. There is also an ongoing debate on whether positive and negative affects should be regarded as related or independent [2, 6]; that is, whether affects should be regarded as bipolar [7] or as separate [8].

Affect has further been described both as a trait and as a state. These two variations can be measured by varying the instruction of the time period the subject is supposed to refer to when filling out an affect scale: for example, *in general, years, weeks, days, today,* or *at the moment* [5, 6]. Schmukle [6] showed trait PA and NA to be two independent dimensions while, in contrast, there was a negative relationship (correlation) between positive and negative state affect. In addition, state affect is reported to be fairly stable over a time period of eight weeks [9], suggesting that state affect is substantially influenced by trait affect.

## 1.2 Affective Personality

Based on the assumption of independence between PA and NA, as measured with the Positive Affect and Negative Affect Scale (PANAS [9];), Norlander *et al.* [10] have described a model for trait “Affective Personality” consisting of four different affective personality types. The four affective personality types are those with high PA and low NA (“Self-actualization”; SA), those with high PA and high NA (“High affective”; HA), those with low PA and low NA (“Low affective”; LA) and those with low PA and high NA (“Self-destructive”; SD). Since the initial description, these personality types have been further characterized [10-12].

SA affective personality is related to a number of advantages, such as better psychological health, with regard to stress and dispositional optimism, as opposed to individuals with an SD affective personality [11]. Studies by Draxler and Hiltunen [30] and Stark and Hiltunen [1] have shown that affective personality can be altered by CBT.

## 1.3 Affect and Psychopathology

Anxiety and depression are associated with state NA, but to a lesser degree with state PA [2, 13, 14]. High state NA has been found in a mixed psychiatric sample [15]. Since there are differences in levels and associations of NA and PA in depression (sub-normal PA and normal NA) and anxiety (high NA and normal PA), it has been suggested that the study of different constellations of affects could contribute to the differentiation between the two conditions [14, 16, 17]. Both anxiety and depression are further related to high trait NA, whereas low trait PA is only related to depression [18-20]. Depressed individuals have a tendency to report low PA and high NA [13, 21] and changes in depression have been shown to be related to changes in trait and state PA and NA in patients during cognitive therapy [18-20]. Kring, Person and Thomas [22] showed that depressive patients in CBT have shown reduced NA during therapy, but no increase in state PA, except in a subgroup of severely depressed patients. Further, a study by Mohr *et al.* [23] showed improvement in state PA in depressive patients after telephone-administered CBT.

## 1.4 Purpose and Hypotheses

As part of an ongoing evaluation of the training therapies at the clinic the aim of the present quasi-experimental study was to investigate how affective personality is related to psychopathology before and after CBT. This is of interest since CBT has been shown to be effective in treating several depressive and anxiety disorders [24]. Further, the hypotheses were (i) that affective personality would be related to psychopathology and, accordingly, personality type would differ between problem areas; (ii) that after therapy, clients would have changed to a healthier affective personality; and (iii) that state PA would increase and state NA would decrease after CBT.

## 2 METHODS

### 2.1 Participants

A total of 73 clients participated in the study, comprising 55 females and 18 males. The participants consisted of self-referred clients receiving CBT at the University psychotherapy clinic. Following assessment phase (2-3 sessions), clients were, depending on individual core-problems, categorized into three problem areas: anxious (*n* = 39), depressive (*n* = 11) or mixed (*n* =23). Since the therapy was problem focused and not syndrome focused, specific diagnoses were not used. Clients took part in 12.3 therapy sessions on average. Demographic and clinical data are described in Table **1**.

## 2.2 Instruments

The PANAS is a self-rating scale used to measure positive and negative affect. It is one of the most utilized affect scales and has been used in many different contexts, such as well-being, psychological distress, mental disorders, stress and social activities [14, 25-27]. Its psychometric properties are reported to be valid and reliable and measures are only moderately affected by demographic variables [6, 8, 9, 15].

The instrument consists of 20 adjectives divided into two 10-item scales measuring positive and negative affect, respectively. Positive affect refers to the extent to which a person feels enthusiastic and active, and negative affect to the extent to which a person experiences anger or guilt [9, 11]. On a five-point scale (from *not at all* to *very much)* the subjects were asked to rate the extent to which they have experienced each specific affect “during the past week.” Subjects, generally, have a tendency to report more positive than negative affect. This tendency is seen across populations, with the exception of psychiatric patients who tend to report more negative affect than non-patients do [9].

Based on exploratory factor analysis and the fact that PA and NA do not correlate significantly, they are assumed to reflect two independent dimensions [9, 15] enabling the construction of four affective personality types. This construction process entails transferring the scale to a diagram, with the PA y-axis corresponding to the NA x-axis, and forming a coordinate system of four squared areas with cut-off points of 35 or lower for low PA, 36 or higher for high PA, 17 or lower for low NA and 18 or higher for high NA [11, 12]. The cut-off points are based on a Swedish norm group (*N* = 1,010) [12].

## 2.3 Procedure

A repeated-measures design was used and clients were measured pre- and post-therapy.

Candidate therapists delivered therapy in their second to fourth semester on the Master’s programme in Psychology with an emphasis on CBT (120 ECTS credits).

Qualified psychotherapists supervised the trainee therapists, and the therapy sessions were recorded by audio or video. Supervisors and the same supervision group could watch/listen to the recordings for educational purposes. Supervision in groups of four trainees was conducted once every third week, 40 hours per term, and each meeting lasted for 3-3.5 hours.

At the beginning of each client contact, the clients together with the therapist signed a client information form stating the rules to be applied (i.e. information about the therapist, therapy process, number of sessions, recordings, confidentiality, supervision and cancellations). Written consent to participate in the evaluation of the activities at the training site was also given by the clients.

As part of this programme, the candidate therapists were provided teaching, training and supervision of behavioural- and functional analysis, and corresponding treatment methods based on textbook descriptions of cognitive and behavioural interventions [28]. Specific psychiatric diagnoses were not generated and the treatment was problem-focused rather than syndrome-focused.

Clients participated in 2-3 evaluation sessions before the beginning of therapy. The grouping of clients (i.e., anxious, depressive and mixed) was based on the target problem that was elucidated during this evaluation and later was the focus of the therapy. Therapy was carried out individually, one session per week over approximately one semester, each session lasting around 45 minutes. The therapy was free of cost and neither clients nor therapists received economic compensation. The clients were asked to fill in the PANAS once during the evaluation sessions and once after the completion of therapy. State affective personality for the last week was thereafter calculated for each individual at both measurements using the cut-off points of Carlstedt *et al.* [12].

## 2.4 Data Analysis

Group differences, effects over time and differences between group changes over time (i.e., Group × Time interaction) regarding PA and NA were tested for significance by two analyses of variance (ANOVA’s, a split-plot design [29];). Bonferroni correction was used to counteract problems of multiple testing when conducting post hoc tests. The significance level was set at p < .017. Comparison of affective personality before and after therapy, distribution of affective personality and problem areas before and after therapy, and distribution of affective personality before and after therapy for the different problem areas were tested for significance with a Pearson's Chi-square Test. The level of significance was set at 5%. Due to missing data, observations from 60 participants were used in analysis of variance, and 70 (pre) or 63 (post) observations in Chi-square analysis.

## 3 RESULTS

### 3.1 Comparison of Affective Personality Before and After Therapy

There was a difference in the frequency of the different affective personality types between pre- and post-therapy measures (*χ^2^* = 8.41, *df* = 3, two-tailed *p* = .04, 99% CI [0.03, 0.04]). Further analysis showed that the change in the distribution was largest for the two most relevant personality types, “Self-actualization” (SA) and “Self-Destructive” (SD) (*χ^2^* = 7.87, *df* = 1, two-tailed *p* = .005, 99% CI [0.006, 0.01]; *c.f.* Table **2**, section “All clients”)

### 3.2 Distribution of Affective Personality and Problem Areas Before and After Therapy

A chi-square analysis showed that there was no significant difference in the distribution of affective personality across problem areas either before (*χ^2^* = 5.78, *df* = 6, two-tailed *p* = .47) or after (*χ^2^* = 7.10, *df* = 6, two-tailed *p* = .32) therapy. The distribution of affective personality types across problem areas is shown in Table **2**.

### 3.3 Distribution of Affective Personality Before and After Therapy for the Different Problem Areas

To further explore potential differences in changes over time in affective personality in relation to problem areas, two of the problem areas were analyzed separately (“Self-actualization” and for “Self-destructive”). These analyses showed a significant difference over time for the mixed group (*χ^2^* = 3.94, *df* = 1, two-tailed *p* < .05; *c.f.* Table **2**, upper part. In addition, this trend was also seen for the anxious group (*χ^2^* = 3.61, *df* = 1, two-tailed *p* = .057). Post-therapy, a higher number of participants were in the SA group and the number of participants in the SD group had decreased. The distribution of affective personality types across problem areas is shown in Table **2**. Results from the chi-square analysis are illustrated graphically in Figs. (**2a** and **b**).

### 3.4 Comparisons Between PA/NA and Problem Areas Pre-Therapy

Comparisons (ANOVA’s) at pre-therapy showed no significant differences between groups regarding PA (*F*(2, 69) = 3.08, *p* = .053) or NA (*F*(2, 69) = 0.31, *p* = .74).

### 3.5 Comparison Between Pre- and Post-Therapy PANAS

**Fig. (1):**
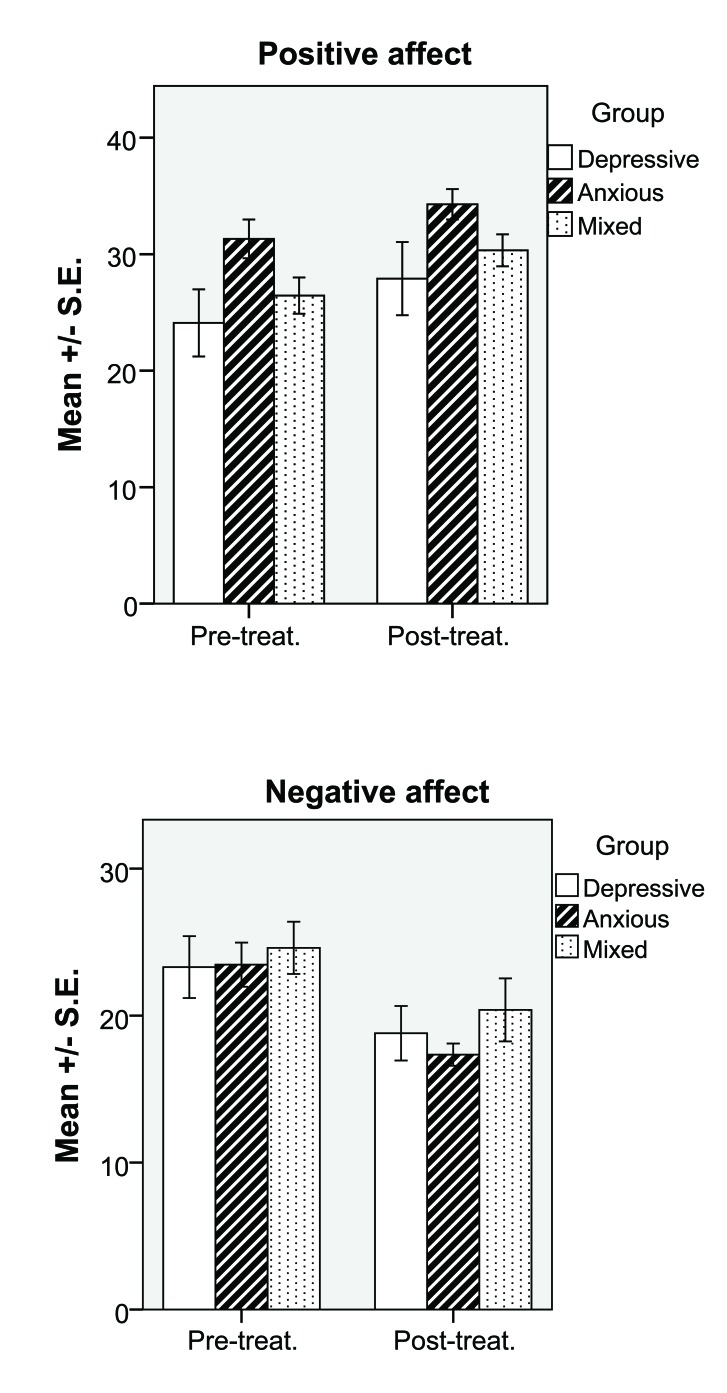
The mean (+ S.E.) of positive and negative affect, respectively, pre- and post-therapy for clients in the Depressive, Anxious and Mixed groups.

**Fig. (2):**
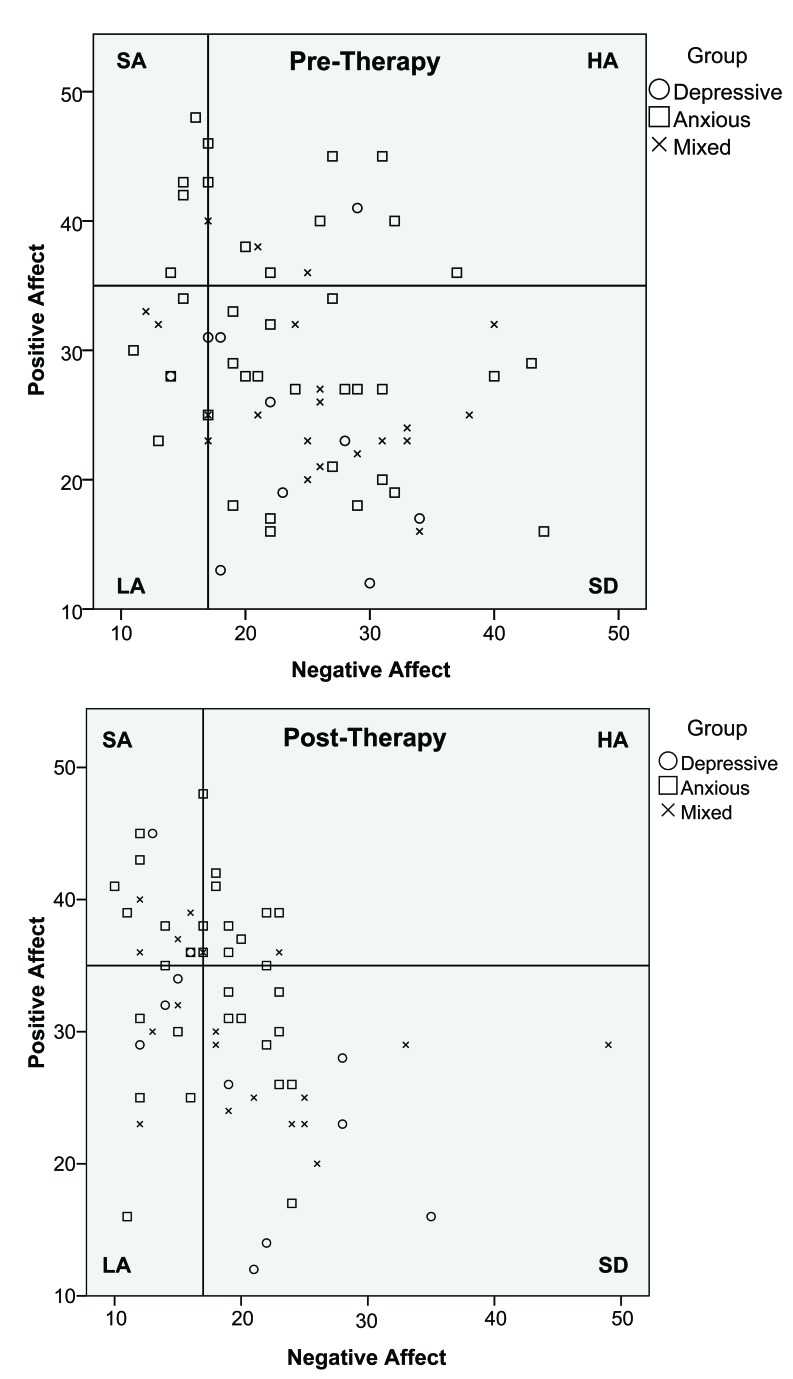
The change of affective personality type over time (pre- and post-therapy) for clients in the Depressive, Anxious and Mixed groups. The four squared areas represent the PANAS dimensions “Self-actualization” (SA), “High affective” (HA), “Low affective” (LA) and “Self-destructive” (SD). The coordinates of the cut-off point are 17 (NA) and 35 (PA).

Analyses of variance of pre- and post-therapy affect showed a difference between pre- and post-therapy measures for both PA (*F*(1, 1) = 5.79, *p* < .02, Cohen´s *d=*0.66) and NA (*F*(1, 1) = 16.73, *p* < .001, Cohen´s *d=*0.98). This is an effect of subjects reporting higher PA and lower NA after, compared to before, therapy (Figs. **1** and **2**).

Further, a significant group effect was found for PA (*F*(1, 2) = 5.83, *p* < .01, Cohen´s *d=*0.86) but not for NA (*F*(1,2) = 0.74, *p* = .48). Post-hoc tests (Bonferroni) showed that this difference was an effect of the anxious group reporting higher PA than the depressive group both pre- and post-therapy (*p* = .01).

There was no significant interaction regarding Group x Time measures of PA (*F*(1, 2) = 0.56, *p* = .95) or NA (*F*(1, 2) = 0.35, *p* = .71).

### 4 DISCUSSION

The aim of the present study was to examine how affective personality is related to psychopathology before and after CBT. Further, the hypotheses were (i) that affective personality would be related to psychopathology and, accordingly, personality type would differ between problem areas; (ii) that after therapy, clients would have changed to a healthier affective personality; and (iii) that state PA would increase and state NA would decrease after CBT.

Overall, results indicated several changes in affect. Clients displayed lower state NA and higher state PA after CBT. At pre-therapy, clients predominantly had an SD affective personality implying high NA and low PA. At post-therapy, there was a significant change in the distribution of affective personality, regardless of problem area, where the number of clients in the SD group decreased while the number of clients in the SA group increased.

### 4.1 Pre-Therapy Affective Personality and Psychopathology

Previous studies have shown that anxiety and depression are associated with NA [2, 13, 14], and high NA has been found in a mixed psychiatric sample [15]. These findings are in accordance with the results of the present study, where most clients experienced high state NA at pre-therapy. Analyses also showed that the anxious group reported higher state PA than the depressive group and mixed group at both measurements, which is consistent with previous studies showing that individuals with anxiety generally experience higher PA than individuals with depression [9, 14, 22]. Further, most clients had a self-destructive affective personality at baseline. At pre-therapy, there were no differences between the problem areas’ affective personality. This indicates that, regardless of problem area, clients predominantly had an SD affective personality implying high NA and low PA.

### 4.2 Post-Therapy Affective Personality and Psychopathology

After therapy, there was a significant change in the distribution of affective personality, regardless of problem area. Post-therapy, fewer clients had a SD affective personality, while the number of clients with a SA affective personality had increased. This indicates that affective personality can be altered through CBT, a finding that is consistent with previous studies by Draxler and Hiltunen [30] and Stark and Hiltunen [1]. SA affective personality is associated with a number of advantages, such as better psychological health, with regard to stress and dispositional optimism, as opposed to individuals with an SD affective personality [11].

However, there was no significant difference in the distribution of problem areas across affective personalities after therapy, which indicates that therapy effect occurred irrespectively of problem area. Future research is needed to establish whether changes in affective personality during therapy are independent of psychopathology.

### 4.3 Pre- and Post-Therapy Affect

Results showed that clients experienced lower state NA and higher state PA after CBT. These results are in agreement with previous research [1, 18, 19, 22, 23, 30]. Additionally, changes in depression have shown to be related to changes in PA and NA in patients during cognitive therapy [20].

Low NA and high PA are correlated with a number of advantages. High NA is related to higher levels of stress, poor psychological well-being and lower self-esteem [35], as well as to poor physical health [36], whereas high PA is correlated with better mental health, sociability, activity, prosocial behaviour, physical well-being and coping [37]. Further studies would be of interest to establish whether the therapy effects (higher state PA, lower state NA as well as changes in affective personality) may have beneficial effects on these areas and if the effects are long-term.

The previous study at the University psychotherapy clinic [1] also evaluated therapy for achieving affectivity change, that is, the CBT provided was evaluated in terms of effectiveness. The results showed a statistical difference between pre- and post-therapy PA and NA, which indicates that the clinic appears to be effective regarding affect change. Previous studies have proven CBT performed by candidate therapist to be effective regarding symptom reduction [32-34] and the present study indicates that candidate therapists can achieve affect change as well.

### 4.4 Limitations

Due to practical and ethical considerations, the study lacks a control group which affects the generalisability of the results. The lack of a control group increases the risk of confounders such as regression to the mean and spontaneous remission. Only a control group could exclude the possibility of these biases and confounders. Statistical methods, such as benchmarking comparisons, are an alternative to a control group or wait list designs. However, benchmarking studies on CBT and PANAS are currently not available. Standardized diagnostic procedures were not used as the therapy provided was problem-focused and not syndrome-focused. Results showed no effects for problem areas across affective personality and reliable diagnoses would have been favourable as differences may have been easier to detect or dismiss. Further, due to the low number of participants in the depressive group the group comparisons may be underpowered. A higher number of participants in this group would have been favourable.

## CONCLUSION

The focus on affects and related areas, such as affective awareness and coping, in relation to CBT have increased over the years [2] and CBT has proven to be effective in obtaining affect change [1, 18, 19, 22, 23, 30]. Low NA and high PA are related to a number of advantages [33, 37] and so is SA affective personality [11]. Contrary to the cross-sectional design used by Stark and Hiltunen [1], a within-subject design was used in the present study, which gives more strength to the indications that affective personality is positively affected by CBT. Results indicate that CBT performed by candidate therapists can achieve changes in affect and affective personality. After therapy, clients experienced higher state PA, lower state NA and an increased number of clients displayed a healthier affective personality.

## Figures and Tables

**Table 1:** Clients’ demographic and clinical data.

Variable	All clients	Depressive	Anxious	Mixed
*n* = 73	*n* = 11 (15%)	*n* = 39 (53%)	*n* = 23 (32%)
Sex (*n* = 73)				
Female	55 (75%)	6 (8%)	30 (41%)	19 (26%)
Male	18 (25%)	5 (7%)	9 (12%)	4 (5%)
Age (years) (*n* = 72)				
Interval	20–51	22-51	20-46	21-48
*M* (*SD*)	29.6 (7.3)	30.7 (8.7)	28.4 (7.1)	30.9 (7.0)
Therapy Sessions (*n* = 71)				
Interval	5–27	5–17	5–27	6–22
*M* (*SD*)	12.3 (4.0)	13.0 (4.4)	12.6 (3.8)	11.5 (4.1)

**Table 2:** Distribution of affective personality across problem areas, and all clients, pre- and post-therapy.

	HA		LA		SD		SA	
	Pre	Post	Pre	Post	Pre	Post	Pre	Post
Depressive	1	0	2	3	7	6	0	2
Anxious	7	7	5	6	20	10	6	10
Mixed	2	1	3	3	16	10	1	5
All clients^a^	10	8	10	12	43^b^	26 ^b^	7 ^b^	17 ^b^

**Note:**HA=High affective; LA=Low affective; SD=Self-destructive; SA= Self-actualization. ^a^Significant difference for all groups in distribution (*p* < .05) pre- and post-therapy. ^b^Significant difference for groups SD and SA in distribution (*p* < .005) pre- and post-therapy.
